# The feasibility of a randomised control trial to assess physiotherapy against surgery for recurrent patellar instability

**DOI:** 10.1186/s40814-020-00635-9

**Published:** 2020-07-06

**Authors:** U. Rahman, E. Gemperle-Mannion, A. Qureshi, C. Edwin, T. O. Smith, H. Parsons, J. Mason, M. Underwood, J. Eldridge, P. Thompson, A. Metcalfe, On behalf of the PIPS study team, Andrea Bailey, Robert Jones, Agnes Hunt, Andrew Barnett, David Beard, Leela Biant, Tarek Boutefnouchet, Jaclyn Brown, Damian Clark, Matt Costa, Loretta Davies, Kerri McGowan, Shilpa Patel, Claire Robertson, Joanna Teuke, Feisal Shah, Benjamin Smith, Nicholas Smith, Tim Spalding, Michael Whitehouse, Celia Wogan, David Wright

**Affiliations:** 1 Warwick Clinical Trials Unit, University of Warwick, Coventry, UK; 2 University Hospitals Coventry and Warwickshire NHS Trust, Coventry, UK; 3 Nuffield Department of Orthopaedics and Rheumatology, University of Oxford, Oxford, UK; 4 Health Economics Department, Warwick Medical School, University of Warwick, Warwick, UK; 5 Bristol Royal Infirmary, University Hospitals Bristol NHS Trust, Bristol, UK

**Keywords:** Patellar instability, Patellar dislocation, Randomised controlled trial, Feasibility study, Patellofemoral, Knee surgery, Physiotherapy, Quality of life, Patient-reported outcome measures

## Abstract

**Background:**

Patellar instability is a relatively common condition that leads to disability and restriction of activities. People with recurrent instability may be given the option of physiotherapy or surgery though this is largely driven by clinician preference rather than by a strong evidence base. We sought to determine the feasibility of conducting a definitive trial comparing physiotherapy with surgical treatment for people with recurrent patellar instability.

**Methods:**

This was a pragmatic, open-label, two-arm feasibility randomised control trial (RCT) with an embedded interview component recruiting across three NHS sites comparing surgical treatment to a package of best conservative care; ‘Personalised Knee Therapy’ (PKT). The primary feasibility outcome was the recruitment rate per centre (expected rate 1 to 1.5 participants recruited each month). Secondary outcomes included the rate of follow-up (over 80% expected at 12 months) and a series of participant-reported outcomes taken at 3, 6 and 12 months following randomisation, including the Norwich Patellar Instability Score (NPIS), the Kujala Patellofemoral Disorder Score (KPDS), EuroQol-5D-5L, self-reported global assessment of change, satisfaction at each time point and resources use.

**Results:**

We recruited 19 participants. Of these, 18 participants (95%) were followed-up at 12 months and 1 (5%) withdrew. One centre recruited at just over one case per month, one centre was unable to recruit, and one centre recruited at over one case per month after a change in participant screening approach. Ten participants were allocated into the PKT arm, with nine to the surgical arm. Mean Norwich Patellar Instability Score improved from 40.6 (standard deviation 22.1) to 28.2 (SD 25.4) from baseline to 12 months.

**Conclusion:**

This feasibility trial identified a number of challenges and required a series of changes to ensure adequate recruitment and follow-up. These changes helped achieve a sufficient recruitment and follow-up rate. The revised trial design is feasible to be conducted as a definitive trial to answer this important clinical question for people with chronic patellar instability.

**Trial registration:**

The trial was prospectively registered on the International Standard Randomised Controlled Trial Number registry on the 22/12/2016 (reference number: ISRCTN14950321). http://www.isrctn.com/ISRCTN14950321

## Key messages regarding feasibility

1. What uncertainties existed regarding the feasibility?
  - People presenting with patellar instability are often adolescents or young adults and are typically younger than participants in most orthopaedic trials. Previous trials have shown that recruiting and retaining this population can be challenging. We did not know whether people in this population would be willing to enter a randomised trial of surgery against non-operative treatment. A method of retaining such young participants in this study was required. The feasibility of delivering the physiotherapy and surgical interventions across multiple face-to-face sessions in the NHS also needed to be assessed. Further uncertainties included the ability to collect data using various patient-reported outcome measures in this population.
2. What are the key feasibility findings?
  - Recruitment and retention of participants were within expected ranges, with adequate recruitment rates at sites after a modification of the design, and retention was very good with 95% follow-up rate at the study primary endpoint. Seventy percent of participants in the physiotherapy intervention reached the minimal compliance level for intervention fidelity. All surgical participants underwent their elected surgical intervention.
  - Participant retention was increased by offering greater flexibility on method of follow-up (i.e. telephone, online, or face-to-face) and by thanking participants for their participation in the study using shopping vouchers. Voucher incentives were successfully adopted for the 12-month follow-up. Some of the secondary outcome instruments were removed based on missing data and interview reports of perceived complexity, which simplified the follow-up questionnaires to ensure better completion of more important measures.
3. What are the implications of the feasibility findings for the design of the main study?
  - This study indicates that the revised trial design is feasible to be conducted as a multi-centre definitive trial. A future full trial would be enhanced by taking on board these changes and by utilising the findings of the interview study. This is warranted to determine the effectiveness of these two current treatment options for patients with chronic patellar instability.

## Background

Patellar instability is a cause of substantial disability and distress in adolescents and young adults [1, 2]. Approximately 50 to 70% of people [3] who have a first-time patellar dislocation will have further or persistent symptoms of patellar instability. This can render otherwise fit and well individuals incapable of continuing their education or work. Patellar instability is typically used to describe a spectrum of symptoms from recurrent frank dislocation to a sensation that the patella is about to dislocate during an activity [4]. Although dislocations are painful, the most disabling problem is often the perception that the patella is moving or about to dislocate leading to activity modification and restriction [4].

Seven out of 100,000 people have a patellar dislocation annually, typically a result of trauma or abnormal patellofemoral morphology. Two thirds (69%) of people with a first-time patellar dislocation are in the second decade of life [5, 6]. It is one of the most common causes of knee injury in adolescents [1]. In this population, the incidence of patellar dislocation is as high as 43 per 100,000 [6]. Half (48%) of those who have a first-time patellar dislocation will go on to have a further episode of dislocation [7]. In those who have a second dislocation, the risk of persistent or recurrent dislocation is even higher [7].

In the UK, recurrent patellar instability is typically managed with a range of non-operative measures or with surgery [8]. The choice between the two is currently based on the opinion of the treating clinician. There is no randomised trial evidence to determine best practice in recurrent instability, and clinicians have to rely on individual clinician judgement supported by case series data, mostly focused on surgery with little evidence on non-operative means [9–12].

Approaches to physiotherapy vary in terms of both the activities undertaken and the length of treatment. A 2011 survey in the UK found wide variability in the non-operative management of patellar instability, from provision of advice and education to individualised treatment plans delivered by expert physiotherapists [13]. Surgical intervention depends on the underlying pathology. A number of narrative reviews have been published in recent years [14–17]. The most commonly used procedure for people suffering with recurrent patellar instability is medial patellofemoral ligament (MPFL) reconstruction. A tibial tubercle osteotomy (TTO) may also be performed, often in combination with MPFL reconstruction, especially in those with patella alta [17]. Case series have suggested good outcomes for MPFL reconstruction, alone or combined with tubercle osteotomy approaches [14–20].

In 2015, a study of people with primary dislocations who had been treated with physiotherapy only demonstrated that patients who did not have surgery or re-dislocation still reported ongoing disability [3, 11]. A 2015 Cochrane review (5 studies, 344 participants, all for first-time dislocation, with no studies in recurrent dislocation) found a lack of evidence supporting either physiotherapy or surgery, concluding there was a need for a randomised control trial comparing the two, especially in recurrent instability where there was no randomised trial evidence [12].

In practice, given the age of those affected [1], performing such a trial presents substantial challenges particularly regarding recruitment and retention in addition to data collection approaches. Further challenges exist around designing and delivering the surgical and physiotherapy intervention protocols. As such, a feasibility randomised control trial was designed to test a randomised controlled trial design, principally to assess methods of recruitment, retention of participants, clinician and patient equipoise (using a variety of quantitative and qualitative measures) and the methods of data collection. This paper reports the result of this feasibility study.

## Methods

### Trial design

#### The patellar instability

Physiotherapy or surgery (PIPS) trial was a two-arm, feasibility RCT with embedded interview component, ahead of a definitive multi-centre RCT evaluating the clinical and cost-effectiveness of surgical intervention compared to physiotherapy for the treatment of recurrent patellar instability. People who presented with recurrent patellar instability to secondary care orthopaedic clinics were approached. Once eligibility had been confirmed and informed consent was obtained, baseline scores were collected and participants were randomly allocated using a 1:1 ratio to a decision to offer Personalised Knee Therapy (PKT), a physiotherapy-led intervention, or a decision to offer surgery. Recruiters, clinicians and patients were all un-blinded to the intervention received. There was no restriction to cross-over from the assigned allocation.

Participants were followed up at 3, 6 and 12 months using a questionnaire pack. This contained a number of patient-reported outcome measures (PROMs) and resource use questions administered via postal and (in the later stages of the study) email and web-based questionnaires. A 6-month interview was performed with participants to ascertain the acceptability of treatment and follow-up methods.

### Participants

#### Eligibility criteria

Our inclusion criteria were aged 16 and over with closed growth plates on MRI scanning (taken as part of standard clinical care) have experienced (self-reported) two or more lateral patellar dislocations or one dislocation with a minimum of a 6-month history of subjective patellar instability leading up to the time of recruitment. Participants must also have been able to give written consent.

Our exclusion criteria were as follows: had another knee condition that resulted in instability symptoms (e.g. cruciate ligament rupture, unstable meniscal tear which has not been treated); had past knee surgery (except for simple arthroscopy with or without lateral release, or previous meniscal surgery); had developmental abnormalities of the lower limb requiring complex surgical intervention, either in the form of severe trochlea dysplasia which, in the opinion of the treating surgeon, required trochleoplasty, or rotational, coronal or sagittal mal-alignment of the femur or tibia which, in the opinion of the treating surgeon, required surgical correction (i.e. osteotomy); previous entry into the trial for the other knee; and had osteochondral defects or chondral injury requiring surgery (including removal of a loose body). Although rare, those with a medial patellar dislocation were also not eligible nor were those who were unable to adhere to trial protocols or complete questionnaires.

#### Settings of care

Three UK centres recruited into the study were as follows: University Hospitals Coventry and Warwickshire (UHCW), The Robert Jones and Agnes Hunt Orthopaedic Centre (Oswestry)/Shrewsbury and Telford (RJAH) NHS Trust and University Hospitals Bristol (UHB) NHS Foundation Trust. The study was not eligible for adoption on the UK Clinical Research Network Portfolio, and screening and recruitment activities were performed on an unfunded basis by site clinical teams, based on local agreements.

### Interventions

#### Personalised Knee Therapy

Given the heterogenous nature of interventions available for the non-operative care of patients with patellar instability, an expert consensus group met on 13 April 2016 to determine the optimal package of non-operative care that would be used in this trial. Participants included five UK physiotherapists, all of whom were senior practitioners with a sub-speciality interest in knee rehabilitation. The meeting was also attended by two knee surgeons with an interest in patellofemoral instability, a behavioural psychologist and academics from the trial team including Oxford, Norwich and Warwick. A systematic review [11] of the non-operative management of patellofemoral instability was presented to the group at the start of the meeting. A package of non-surgical care designed by the team for the trial was agreed upon detailing the principles of treatment and referred to as ‘Personalised Knee Therapy’ (Appendix 1).

Participants randomised to this arm were referred by the surgical team to the physiotherapy team at the participating centre for delivery of PKT. The interventions were provided by any qualified physiotherapist over a planned six sessions but if clinically required, PKT could be performed over more sessions if the treatment aims were met after six sessions.

A treatment booklet was developed for PKT. This included physiotherapist instructions and a case report form combined with clinical notes that could be used in the clinical record, to make trial-related recoding of the interventions easier for physiotherapists. This allowed physiotherapists to record the advice and interventions given to participants in the PKT group at each session, as well as reporting the length of the session, key aims of the interventions, home exercise instructions and complications.

#### Surgery

Participants recruited to the surgical arm were offered elective surgery. The intervention to be undertaken was based on the individual surgeon’s decision and on the participant’s clinical presentation. Approaches and techniques for the operation were determined by each surgeon. A standard form was designed that allowed surgeons to record the type of operation performed, complications and post-operative instructions.

To define the proposed surgical intervention for a potential main trial, a surgical consensus meeting took place on 28 November 2017, involving eight consultant surgeons with an expressed interest in the treatment of patellofemoral joint disorders. In this meeting, the current literature was discussed and an algorithm was developed for the surgical management of patellofemoral instability suitable for use in the trial. The findings of this meeting were recorded and subsequently used to help develop a national guideline for the surgical management of patellar instability, which has completed a national consensus process and is planned for publication this year.

Each participant was referred for a standard package of physiotherapy after surgery as determined by the same consensus group for the non-surgical arm. This was a distinct package of care from PKT (Appendix 2).

### Sample size

As this was a feasibility study, no formal power calculation was needed. A maximum target of 50 participants was set, corresponding to expectation that each site would recruit 1 to 1.5 participants per month over the 12-month recruitment period. However, if the engagement rate was lower than planned, recruitment would be ended 12 months after the recruitment of the first participant. Potential participants who had previously been approached and received study information could be recruited for up to 3 months after this time point.

### Randomisation

Randomisation was performed using an independent telephone-based randomisation service at Warwick Clinical Trials Unit (WCTU). Participants were randomised strictly sequentially using a 1:1 randomisation ratio, stratified by joint hypermobility (defined by a Beighton’s [21] score of four or more) or the presence of patella alta, (defined by a Biedert [22] ratio of < 0.25 on a sagittal MRI scan as determined by the treating clinician), using a random block size of four or six. The randomisation list was prepared by the study statistician (HP) who had no contact with participants throughout the study.

### Outcomes

The primary outcome was the recruitment rate per study recruitment centre. Secondary outcomes collected were as follows: follow-up rates, the Norwich Patellar Instability Score (NPIS) [23], the Kujala Patellofemoral Disorder Score (KPDS) [24], the Banff Patellar Instability Instrument (BPII) [25] and EuroQol-5D-5L [26], self-reported global assessment of change and satisfaction at each time point and resources use. These participant-reported measures have been validated for use in patients with patellar instability [27, 28]. The NPIS (scored 0–100, with 0 being best score possible) and KPDS (scored 0–100, with 100 being best score possible) were scored according to their respective instructions whilst the EQ-5D-5L was scored using crosswalk values to the UK EQ-5D-3L dataset [29].

At the 3- and 6-month follow-up, poor follow-up rates were noted by the Trial Management Group (TMG), and a number of changes were made. Given the extremely low completion rate for the BPII at the 3-month follow-up and interviews suggesting it was complex to understand and fill in, it was dropped from the 6-month questionnaire onwards for all participants and will not be reported in this paper; this reduced the size of the questionnaire substantially. A web-based, mobile-enabled questionnaire was developed which was implemented after the 3-month follow-up. Participants could choose to either complete the web-based form or a paper form. Participants were given a £20 Amazon voucher at study end for their participation, regardless of follow-up, and this was sent with the final questionnaire posting at 12 months.

### Interview component

Telephone interviews were offered for all participants at the 6-month time point. They were anonymised and transcribed by an external agency (TypeOut, Surrey, UK) and stored in a secure online database. Thematic analysis was performed on each participant’s interview transcript to determine overall narratives in the responses. Data was hand sorted and codes were assigned to the data, before an analysis of the overarching themes was conducted. Key themes were then collated and reported.

### Statistical analysis

Results were collated regarding the frequency of study feasibility measures. As this was a feasibility study, it was not the intention to provide a comparison of the two intervention arms. PROMs are presented for the whole population only. In order to ensure there was not a major safety issue in either of the interventions, complications were examined for separate allocation groups. Between-group data are presented on an intention to treat basis (i.e. as randomised, regardless of crossover). Analyses were performed using SPSS V25.0 (IBM, USA) and Microsoft Excel (Microsoft, USA).

## Results

### Recruitment

Recruitment started in March 2017 and closed in May 2018 at all centres. Follow-up was completed in May 2019 for all participants, and the study was closed in June 2019 as planned.

**Fig. 1:**
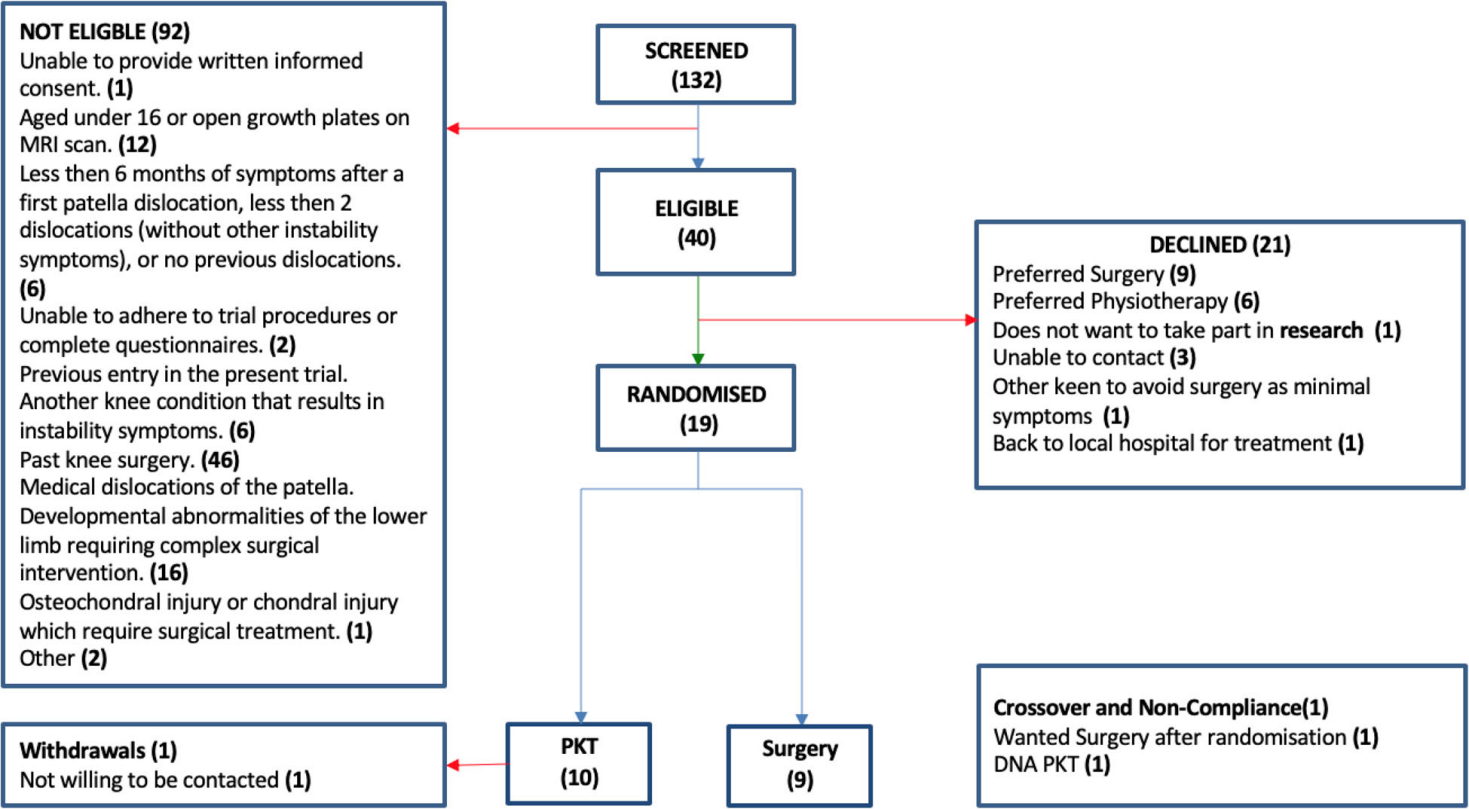
CONSORT participant flow

We screened 132 people for eligibility across the 3 sites; 92 were deemed ineligible as per the a priori exclusion criteria, and 46 had had previous surgery on the study knee. Sixteen had chondral injury on the routine pre-consultation MRI and were referred directly for surgery; a further 16 had open growth plates on MRI; and further 14 failed to meet the eligibility criteria for a range of reasons (Fig. 1).

Forty potential participants were deemed eligible for the study and counselled on participation; 21 declined invitation to take part with 9 patients preferring surgery over physiotherapy and 6 preferring physiotherapy; a further 6 declined invitation to participate (Fig. 1).

Nineteen participants were recruited into the study with 10 in the PKT arm and 9 in the surgery arm. Recruitment charts for the three centres are in Appendix 3. One centre, in which recruitment was undertaken across all active fracture clinics and knee clinics in the Trust with full-time research nurse support, recruited consistently at or just above one participant per centre, per month. A second centre, a highly sub-specialised tertiary referral centre with research nurse support, was unable to recruit to the study. In this latter site, there were a very high number of people with open growth plates or requiring trochleoplasty, and of those people who were suitable, all preferred surgery. The third centre, also a highly sub-specialised tertiary referral clinic but without research nurse support, did not recruit at first but then instituted a number of changes, including a research doctor in clinic to help recruitment, and also informing the Emergency Department and fracture clinic staff referring into the clinic about the study. This substantially improved recruitment, the site recruited at a rate of two participants per month after this intervention.

### Baseline characteristics

The mean age of the 19 participants was 26 (SD 12.0) years, with 8 (42%) males and a mean BMI of 28 (SD 8). The mean age at first patellar dislocation for study knees was 19 (range 7 to 39 years, inter-quartile range 8 years) and 5 participants (26%) had bilateral patellar instability, though only data from one knee was included the study and as per the protocol the most symptomatic side was included for these people (Table 1).

**Table 1:** Baseline characteristics and scores (*n* = 19)

Patient variable	Number
*Number randomised (n)*	*19*
Age (in years; median, range, IQR)*	24, 16–48 (11)
Gender (male: *n*, %)	8 (42)
BMI (mean, SD)	29 (8)
Study knee (left; *n*, %)	7 (38)
Number of dislocations in study knee (median, IQR)	4 (3)
Bilateral disease (*n*, %)	5 (26)
Number of dislocations in non-study knee (median, IQR)	2 (1)
Age at first knee dislocation (years; range, IQR)	7-39 (8)
Beighton’s Score of study knee (mean, SD)	4 (3)
Joint hypermobility in study knee (Beighton’s Score ≥ 4: *n*, %)	7 (38)
Biedert patella–trochlea index of study knee (mean, SD)	0.3 (0.1)
Patella Alta in study knee (Biedert patella–trochlea index < 0.25: *n*, %)	3 (16)
Previous physiotherapy in study knee (any; *n*, %)	18 (95)

*Calculated to randomisation date into study

The mean Biedert Patella-Trochlea Index was 0.3 (SD 0.1), whilst the Beighton score was 3.9 (SD 2.9). Seven participants (38%) had joint hypermobility (defined as a Beighton Score equal or greater than four). Eighteen (95%) participants had received prior physiotherapy.

### Outcomes

#### PROMs

**Fig. 2:**
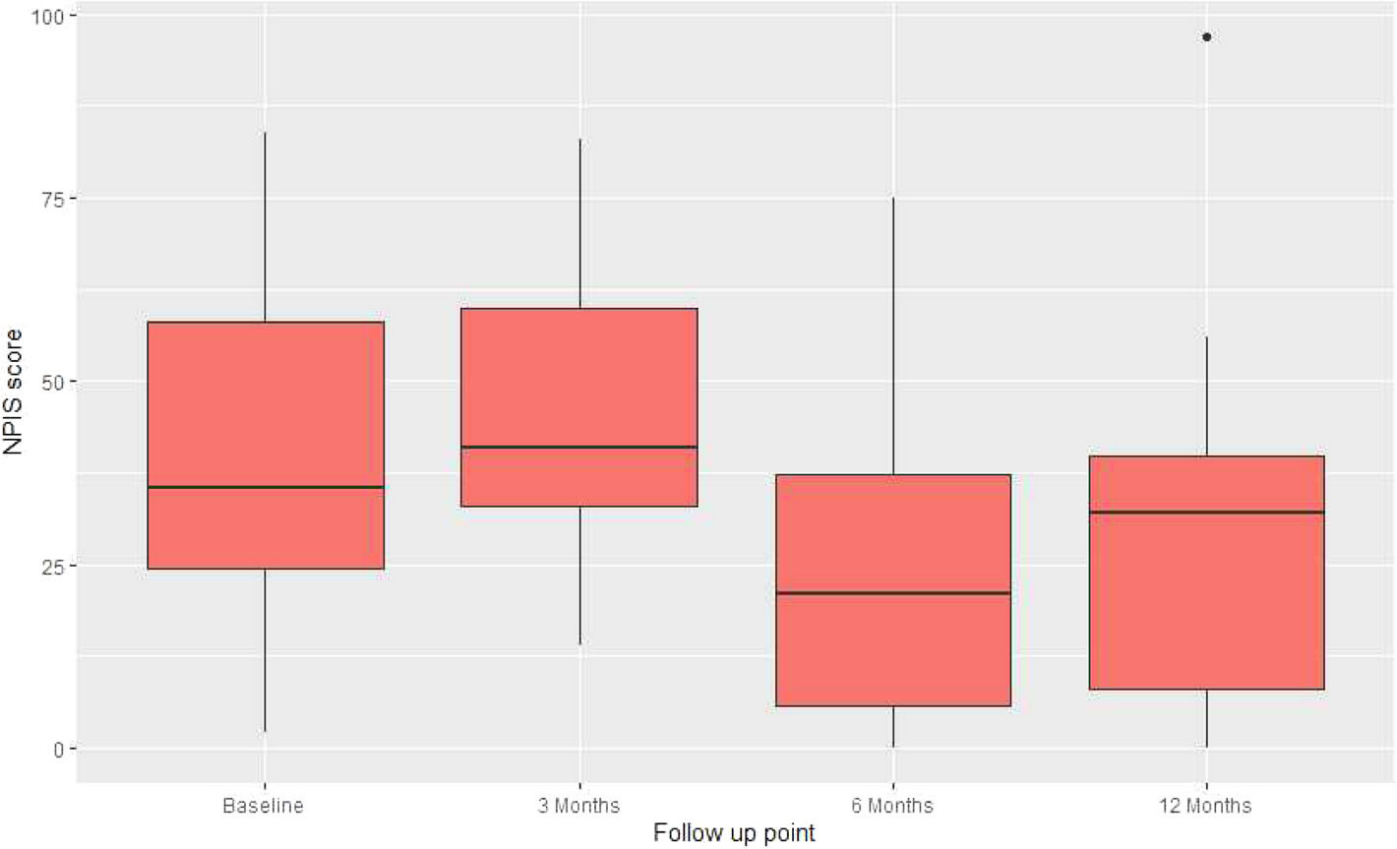
Box and whisker plot of NPIS for entire cohort by time point

Eighteen participants (95%) completed the 12-month follow-up questionnaire. Fewer participants completed the 3- and 6-month follow-up questionnaires each with 15 (79%) and 12 (63%) responders, respectively (Table 2). Once this was recognised, the team made a number of interventions to improve follow-up, as noted in the “Methods” section. Consequently, follow-up rates improved substantially, to the point that we obtained complete 12-month dataset on 18 of 19 participants. Of those offered the online questionnaires by e-mail, two (20%) and six (32%) responses were received this way at 6 months and 12 months, respectively (Fig. 2).

**Table 2:** Follow-up rate and methods of response

Follow-up outcome	Baseline	3 months	6 months	12 months
Number of valid responses at time point	19	15	12	18
Offered paper based CRF	19	19	18	18
Offered web-based CRF	0	0	10	18
Completed web-based CRF	0	0	2	6
Completed postal CRF	19	15	10	12

The NPIS for the cohort improved from a mean score of 40.6 (SD 22.1) to 28.2 (SD 25.4) at 12-month follow-up. Similarly, the KPDS improved from 62.1 (SD 19.3) to 79.8 (SD 14.8) and the EQ-5D-5L from 0.65 (SD 0.25) to 0.82 (0.13) (Table 3 and Fig. 2).

**Table 3:** Patient-reported outcome measures

Follow up outcome		Baseline	3 months	6 months	12 months
NPIS (mean, SD) (0=lowest possible function, 0=best possible function)		40.6 (22.1)	47.1 (18.8)	25.2 (23.7)	28.9 (24.8)
KPDS (mean, SD) (0=lowest possible function, 100=best possible function)		62.1 (19.3)	61.8 (18.6)	73.1 (16.3)	79.4 (14.4)
EQ5D (mean, SD) (0=worst health, 1=perfect health)		0.65 (0.25)	0.61 (0.28)	0.79 (0.15)	0.82 (0.82)
Global assessment of change (n, %)	Substantially Better	-	1 (5)	6 (32)	8 (42)
	Moderately Better	-	6 (32)	2 (11)	5 (26)
	No difference	-	6 (32)	4 (21)	3 (16)
	Moderately worse	-	1 (5)	0 (0)	2 (11)
	Substantially worse	-	1 (5)	0 (0)	0 (0)
Satisfaction with treatment (n, %)	Extremely Satisfied	-	2 (11)	7 (37)	8 (42)
	Very Satisfied	-	4 (21)	2 (11)	3 (16)
	Somewhat Satisfied	-	5 (26)	0 (0)	3 (16)
	Neither satisfied nor dissatisfied	-	2 (11)	2 (11)	3 (16)
	Somewhat Dissatisfied	-	1 (5)	1 (5)	1 (5)
	Very Dissatisfied	-	1 (5)	0 (0)	0 (0)
	Extremely Dissatisfied	-	0 (0)	0 (0)	0 (0)

The self-reported global assessment of change 5-point scale showed that the majority of participants improved during follow-up (Table 3). Eight participants (42%) reporting feeling ‘substantially better’ at 12-month follow-up compared with only one participant (5%) at 3 months. Likewise, a satisfaction score was taken using a 6-point scale showing eight participants (42%) were ‘extremely satisfied’ at 12 months compared with only two (11%) at 3 months.

Complication data was also collected (Table 4). This included data on muscle soreness, re-dislocation of the study knee and ankle or hip pain. The overall incidence of complications increased through the follow-up period from 47% at 3 months to 68% at 12 months. The majority of these complications may be considered relatively minor including muscle soreness or hip/ankle pain which may be considered a normal part of recovery from either physiotherapy or surgery. No safety concerns were reported at any point. Patellar dislocation rates within the study stayed consistent between time points ranging from 16% (*n* = 3) and 11% (*n* = 2) at different time points, overall 6 individuals had a patellar dislocation during the study period. Those with ankle, hip and knee pain increased as well as those with muscle soreness over time.

**Table 4:** Complication data, whole cohort and by allocation

Follow up outcome	3 months			6 months			12 months		
	PKT	Surgery	Whole Cohort	PKT	Surgery	Whole Cohort	PKT	Surgery	Whole Cohort
Any complication (n, %)	6 (32)	3 (16)	9 (47)	3 (16)	3 (16)	6 (32)	6 (32)	7 (37)	13 (68)
Complication: Study Knee Patellar Dislocation (n, %)	3 (16)	0 (0)	3 (16)	1 (5)	1 (5)	2 (11)	1 (5)	1 (5)	2 (11)
Complication: Injury (n, %)	1 (5)	0 (0)	1 (5)	0 (0)	1 (5)	1 (5)	0 (0)	2 (11)	2 (11)
Complication: Muscle soreness (n, %)	4 (21)	1 (5)	5 (26)	2 (11)	2 (11)	4 (21)	4 (21)	4 (21)	8 (42)
Complication: Ankle or hip pain (n, %)	1 (5)	2 (11)	3 (16)	0 (0)	3 (16)	3 (16)	3 (16)	1 (5)	4 (21)
Complication: DVT or PE (n, %)	0 (0)	0 (0)	0 (0)	0 (0)	0 (0)	0 (0)	0 (0)	0 (0)	0 (0)
Complication: Other (n, %)	1 (5)	0 (0)	1 (5)	2 (11)	1 (5)	3 (16)	3 (16)	3 (16)	6 (32)

Participants also reported whether they were absent from work during the study as a result of the patellar instability. Whilst more people were working at the end of the study, 14 (74%) compared with 4 (21%) at baseline, the number of participants taking time off work did not show any pattern and varied during follow-up.

In the surgical arm, all participants completed intervention by the end of the study with nine patients undergoing medial patellofemoral ligament (MPFL) reconstruction and a further four of the nine also had a tibial tubercle osteotomy (TTO). Four participants in this arm also had a different procedure as determined by the surgical team.

The median time to intervention in the PKT arm was 8 weeks (range 1–53 weeks, IQR 30 weeks); compared with a median of 16 weeks for the surgery arm (range 10–44 weeks, IQR 5 weeks).

One participant was randomised to PKT but shortly after randomisation decided to have surgery. In the PKT arm, one participant did not receive any intervention, and two did not complete the intervention (attended fewer than three sessions). The median number of sessions in the PKT arm for the remainder was five sessions. One participant withdrew after completing the PKT intervention because they did not want to receive further contact but was included in the analysis of baseline and follow-up data to the point of withdrawal as per the study protocol. One further participant was non-compliant with attendance at PKT but did complete the 12-month follow-up questionnaire. All remaining participants completed follow-up at 12 months.

An analysis of hospital records was recommended by the TMG after two surgeons noted that some patients in the PKT arm had subsequently required surgery after their 12-month follow-up (participants had been consented for this activity at the start of the study). This was performed in July 2019 at a median 96 weeks follow-up (range 68–120 weeks, IQR 34.2 weeks). This found that five of those in the PKT arm proceeded to be either listed or have subsequent surgery (for the same knee in the study) for ongoing instability. Of the 10 in the PKT arm, 1 withdrew and further analysis of hospital records was not possible; therefore, 5 out of the 9 of those followed up went on to be listed for surgery, or have surgery, following PKT.

### Interview findings

Interviews with participants revealed that, on the whole, our questionnaires were appropriate both in terms of the relevance to the condition and in length. However, some did complain of the BPII being too long whilst others told of the difficulty in understanding specific questions for this measure. A commonly held view was that questions were repetitive across different PROMs. Those with longstanding instability expressed a bias to wanting surgery regardless of which group they were allocated to. It was seen that physiotherapy was a temporising measure and participants felt that they had been ‘through the process of physiotherapy before’. Most were happy with the treatment they received and displayed a positive attitude towards involvement in research. The web-based questionnaires were received well, with interviews revealing that they reduced the need to actively return the questionnaire by post, and also allowed them to be completed more quickly.

## Discussion

The results of this feasibility study indicate that the revised trial design is feasible to be delivered as a definitive trial. Revisions to the design such as reconsidering recruitment pathways and changing the follow-up to a more multi-modal approach resulted in clear improvements in the delivery of the study.

Whilst the results showed that the recruitment of participants from a young adult and adolescent population is possible, we have identified key difficulties in recruiting this population from different settings. For example, almost all the people screened at the second centre were not eligible for participation, and those that were eligible opted for surgery. The recruitment activities happened at a specialist tertiary clinic where patients are usually referred after treatment at other centres, meaning that more typical patients would not necessarily have been seen there. Site three demonstrated that this problem could be overcome by communication with all people in the trust involved in the identification and referral of such patients, and when a trust-wide coordinated approach to recruitment was taken, recruitment improved substantially.

A future study should focus on recruiting from the places people present to with recurrent patellar instability (such as fracture clinics or general elective clinics), and not only tertiary referral clinics. For the study to be generalisable to the breadth of UK practice, both secondary and tertiary care need to be involved, but recruitment activities should consider the full treatment pathway (from first presentation with recurrent instability) for recruitment to succeed across all settings.

The recruitment for the overall study failed to meet the intended target of 1 to 1.5 participants per centre per month. However, if we exclude the results from site two, other centres met the target rate. More than half the patients approached at those sites were willing to take part, a good rate of recruitment for a trial of surgery against a non-surgical treatment. These provide promising results that it would be feasible to recruit participants to this trial design if conducted as a definitive trial.

Another issue in the recruitment centred on eligible potential participants declining recruitment with almost half of those declining participation because they preferred one treatment option over another. The relative even balance between those who preferred physiotherapy and those who preferred surgery suggests that trial materials were well presented and appropriately communicated the position of equipoise. Certain patients may have had prior advice or prior experience with treatment, particularly those who may have had little benefit from community-based physiotherapy and would want to opt for a perceived more active treatment choice, such as surgery. Equally, those with no prior experience of treatment may have apprehensions about surgery, declining outright and thereby excluding themselves from recruitment.

Regarding the retention of participants, we have been able to show good retention and, particularly at the 12-month follow-up, effective engagement with the trial. This is encouraging as a young study population such as this can prove difficult to follow-up. This was not without issue however, as the trial management group helped to adapt the questionnaires and follow-up method and schedule based on previous responses throughout the study. The development of the online questionnaire helped to engage far more participants than may have been possible with paper questionnaires alone, with one third of the cohort choosing to respond this way at 12 months. Additionally, other measures such as removing certain PROMs from the initial baseline questionnaire proved helpful, as did the gift voucher to recompense people for their participation in the trial. Whilst we do not know which aspect of the change in follow-up process improved retention, the improved response rate at 12 months compared to 3 and 6 months shows that good rates of follow-up can be achieved in this study population with meticulous follow-up processes appropriate to this population.

Participant interviews reported that the majority of people were willing to engage with treatments. Although an overall preference for surgery was expressed, the screening data suggested that similar numbers of people who did not take part did so because they opted for physiotherapy as opposed to opting for surgery. Engagement in PKT was good, just one participant withdrew from PKT citing a lack of desire to engage with the intervention. In a future trial, further modifications to this intervention would be recommended (which were not available with our trial funding) including paper and electronic resources for participants to improve adherence and engagement.

One of the challenges of a future study would be the potential heterogeneity of the PKT intervention. In a main trial, we would recommend a high-quality training plan for research sites and physiotherapists specifically, and good-quality monitoring of the fidelity of the intervention and participant’s interactions with it. Future investigators of a full trial may also consider whether our eligibility criteria or stratification factors were optimal, including the number of prior procedures allowed, or the use of TT-TG to stratify the population or be included in factors in the final analysis. However, overall, we believe that we have pragmatically captured the population for whom this question is relevant, so a generalisable answer can be produced for patients suffering from this challenging problem.

Although the study was able to show that the recruitment and retention of participants was possible for this cohort of patients, there exist a number of key limitations. Firstly, one centre was unable to recruit any participants given the issues highlighted above, although site three demonstrated this could be resolved with appropriate intervention. Additionally, a number of participants were unable to complete the questionnaires when they received them due to difficulty with the questions and also the length of the initial version of the questionnaire. This was largely remedied by the end of the study. However, certain sections of the questionnaire on resource use (e.g. use of medications, use of non-study health resources such as general practitioner consultations) were poorly completed, resulting in a limited yield of information to inform future design. Although it is not known if this was simply because people did not use such resources; a different format of questions may have resolved this. Furthermore, given the finding that half of those in the PKT arm went on to have further surgery for the same knee in the second year after randomisation, it could be argued that the follow-up period was too short and may need to be increased to adequately capture such data.

## Conclusion

A multi-centre randomised trial comparing intensive, consensus-driven ‘best care’ physiotherapy to surgery for the treatment of patellar instability is likely to be feasible in the UK, with an appropriate design. This feasibility study has identified important limitations but also solutions to ensure a future definitive trial could be successfully delivered. We remain convinced that such a study is required given the paucity of the evidence base that exists for the treatment of this relatively common and disabling condition.

## Supplementary information

**Additional file 1.** Supplementary information

## Data Availability

The datasets used and/or analysed during the current study are available from the corresponding author on reasonable request.

## Abbreviations

PIPS: Patellar Instability Physiotherapy or Surgery
MPFL: Medial patellofemoral ligament
TTO: Tibial tubercle osteotomy
RCT: Randomised clinical trial
WCTU: Warwick Clinical Trials Unit
UHCW: University Hospitals Coventry and Warwickshire
RJAH: Robert Jones and Agnes Hunt Orthopaedic Centre
UHB: University Hospitals Bristol
PKT: Personalised Knee Therapy
NPIS: Norwich Patellar Instability Score
KPDS: Kujala Patellofemoral Disorder Score
BPII: Banff Patellar Instability Score
TMG: Trial Management Group
EQ-5D: EuroQol 5D
PROM: Patient-reported outcome measure
