# Supplementary material for: The feasibility of a randomised control trial to assess physiotherapy against surgery for recurrent patellar instability
Source: Pilot Feasibility Stud. 2020 Jul 6;6:94. doi: 10.1186/s40814-020-00635-9 (PMC7336411; doi:10.1186/s40814-020-00635-9)
Supplement: Supplementary file 1 — Additional file 1. Supplementary information [file 40814_2020_635_MOESM1_ESM.docx]

The feasibility of a randomised control trial to assess physiotherapy against surgery for recurrent patellar instability

# Appendices

## Appendix 1 – PKT Principles

##
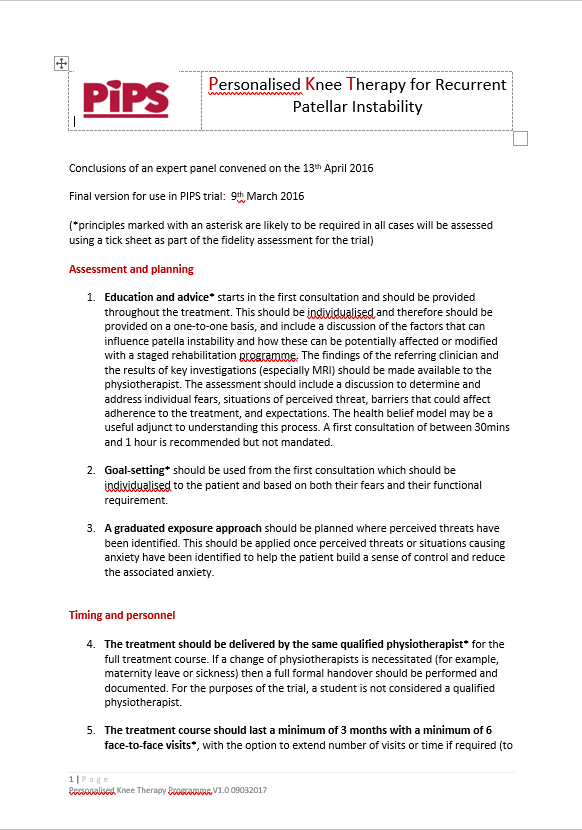


##
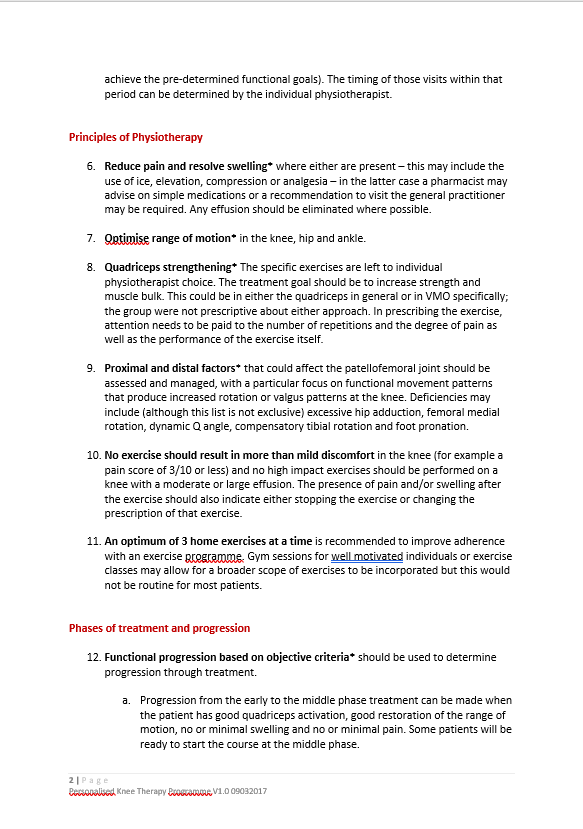


##
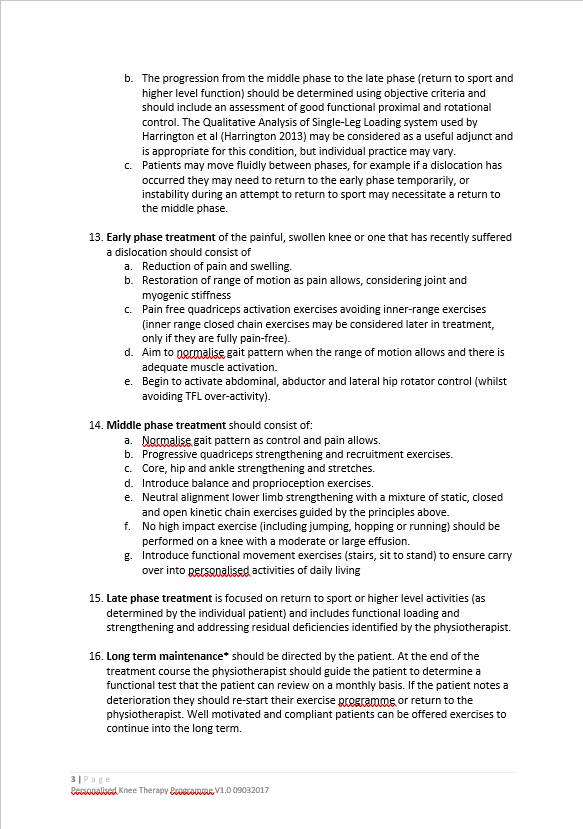


##
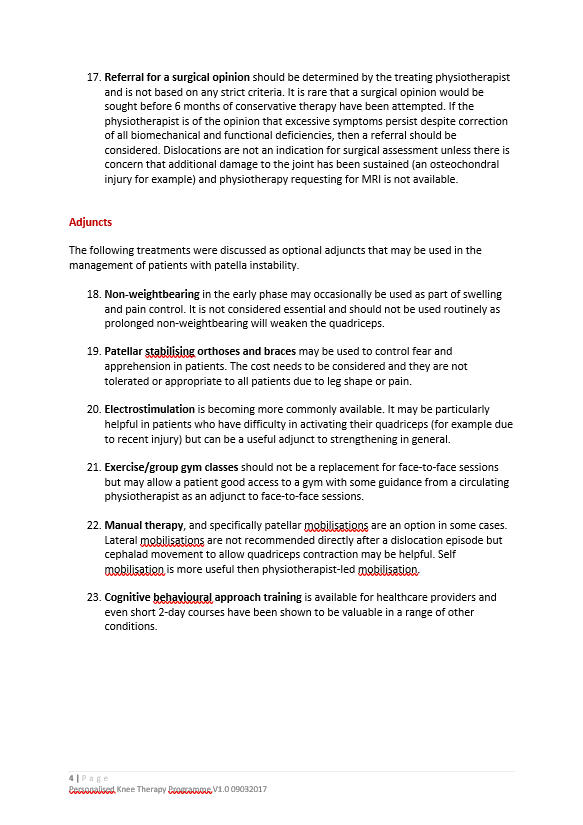


## Appendix 2 – Post-Operative Physiotherapy Principles


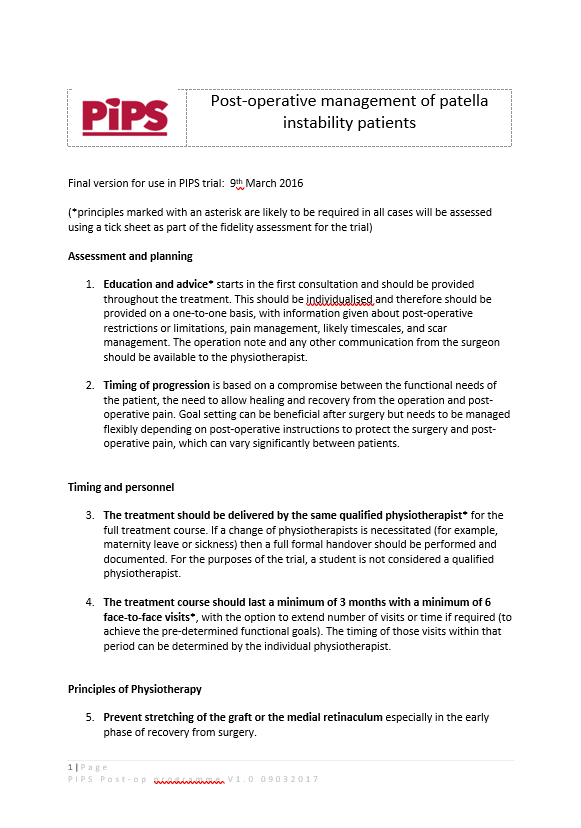


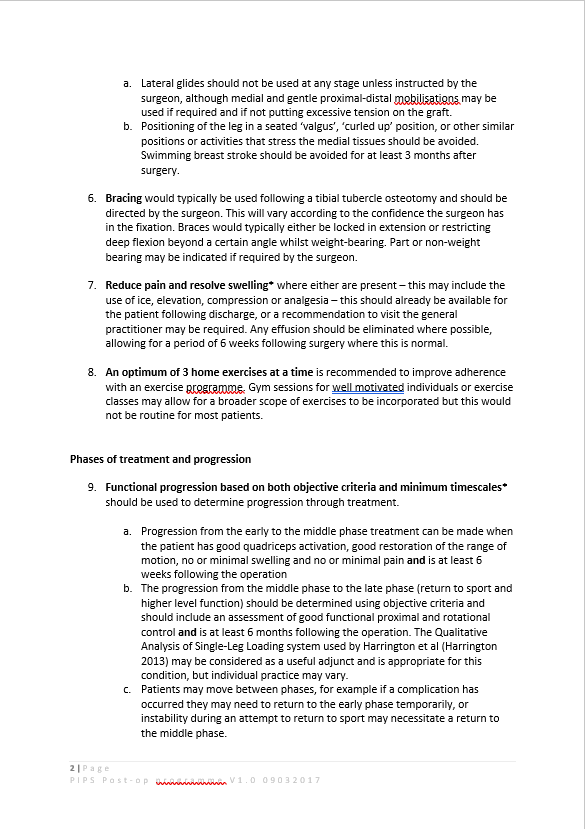


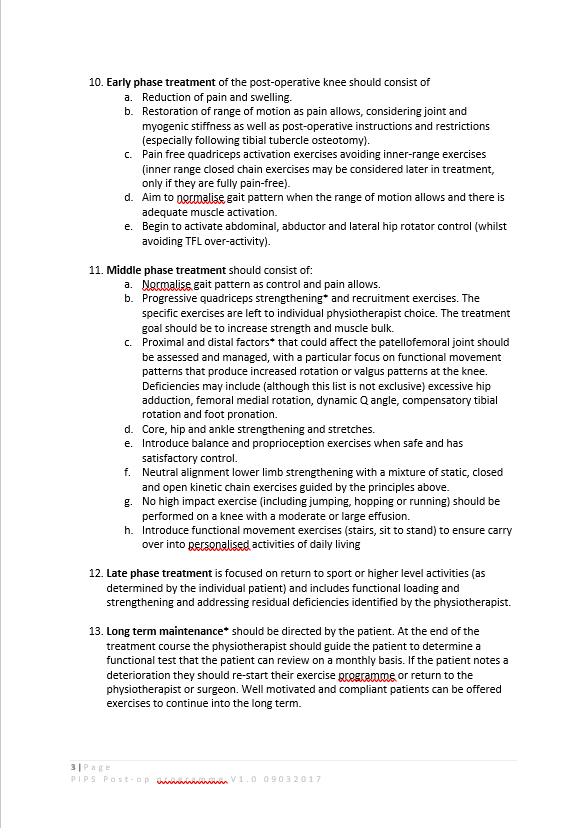


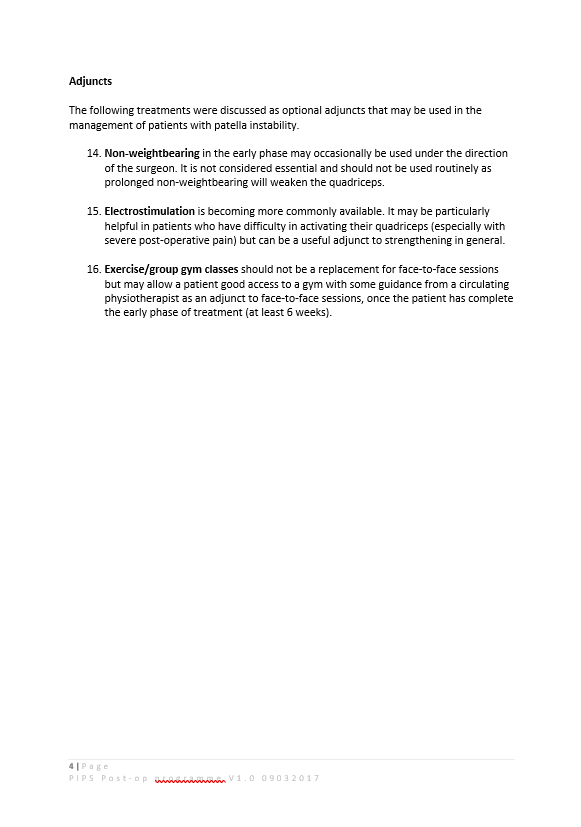


## Appendix 3 – Recruitment Charts By Site

| **Screening variable** | **UHCW** | **Oswestry/ Shrewsbury** | **Bristol** | **All sites** |
| --- | --- | --- | --- | --- |
| Number of patients approached | 29 | 96 | 7 | 132 |
| Number ineligible | 2 | 90 | 0 | 92 |
| Number of patients eligible | 27 | 6 | 7 | 40 |
| Number consented to take part | 14 | 0 | 5 | 19 |
| Number randomised | 14 | 0 | 5 | 19 |

1: Screening & Recruitment per site


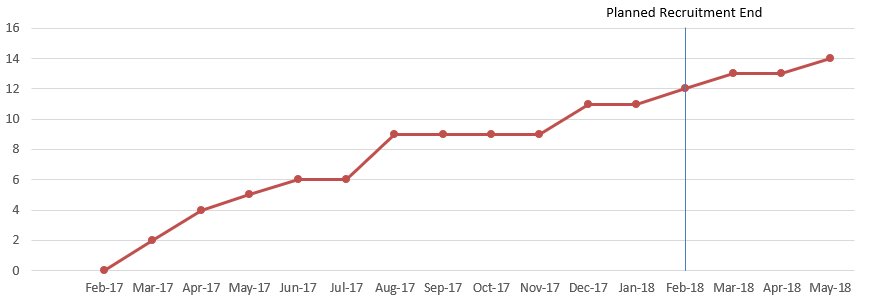


2: Site One Recruitment Chart


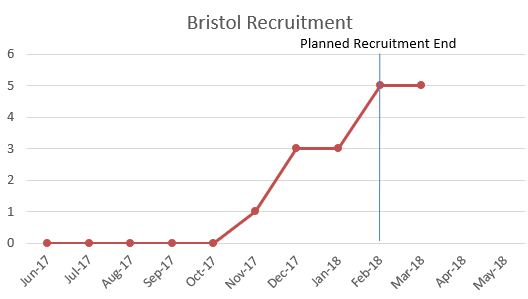


3: Site Three Recruitment Chart
